# YAP is a critical oncogene in human cholangiocarcinoma

**DOI:** 10.18632/oncotarget.4043

**Published:** 2015-05-08

**Authors:** Tiemin Pei, Yuejin Li, Jiabei Wang, Huanlai Wang, Yingjian Liang, Huawen Shi, Boshi Sun, Dalong Yin, Jing Sun, Ruipeng Song, Shangha Pan, Yu Sun, Hongchi Jiang, Tongsen Zheng, Lianxin Liu

**Affiliations:** ^1^ Key Laboratory of Hepatosplenic Surgery, Ministry of Education, Department of General Surgery, The First Affiliated Hospital of Harbin Medical University, Harbin, China; ^2^ Department of General Surgery, Qiqihaer City Hospital of Traditional Chinese Medicine, Qiqihaer, China

**Keywords:** cholangiocarcinoma, YAP, gankyrin, AKT, tumorigenesis

## Abstract

Yes-associated protein (YAP), a transcriptional co-activator, has important regulatory roles in cell signaling and is dysregulated in a number of cancers. However, the role of YAP in cholangiocarcinoma (CCA) progression remains unclear. Here, we demonstrated that YAP was overexpressed in CCA cells and human specimens. High levels of nuclear YAP (nYAP) correlated with histological differentiation, TNM stage, metastasis and poor prognosis in CCA. Silencing YAP increased tumor sensitivity to chemotherapy and inhibited CCA tumorigenesis and metastasis both *in vivo* and *in vitro*. YAP overexpression *in vivo* and *in vitro* promoted CCA tumorigenesis and metastasis. Additionally, we found that YAP induced epithelial-mesenchymal transition (EMT) and formed a regulatory circuit with miR-29c, IGF1, AKT and gankyrin to promote the progression of CCA. Results of CCA tissue microarray showed positive correlations between nYAP and gankyrin or p-AKT expression. Combination of nYAP and gankyrin or p-AKT exhibited improved prognostic accuracy for CCA patients. In conclusion, YAP promotes carcinogenesis and metastasis by up-regulating gankyrin through activation of the AKT pathway.

## INTRODUCTION

Cholangiocarcinoma (CCA) is a devastating cancer originating from the cholangiocytes of the intra- and extrahepatic biliary tract system [1]. Because of the high malignancy of this tumor, the 5-year survival rate is low and prognosis is dismal [2]. As reported, the incidence and mortality of CCA have increased rapidly in recent years [3]. As CCA patients do not benefit much from the adjuvant therapy, including systemic chemotherapy and radiotherapy, surgical resection is identified as the only curative option [4]. However, CCA is always discovered at an advanced stage, which breaks the possibility of curative surgery [5]. Therefore, it is critical to elucidate the molecular mechanisms regulating CCA tumor progression to find potential therapeutic strategies.

Yes-associated protein (YAP), a direct downstream effector of the tumor suppressive Hippo pathway, has been identified as a transcriptional co-activator that interacts with TEA domain family member (TEAD), SMAD family member and other transcription factors to regulate the expression of target genes [6–10]. YAP has been found to be elevated in several types of malignant tumors, such as liver cancer [11], medulloblastoma [12], colon cancer [13], oral squamous cell carcinoma [14], ovarian cancer [15], urothelial carcinoma of the bladder [16], gastric cancer [17], colorectal cancer [18], uveal melanoma [19], and non–small-cell lung cancer [20]. YAP activation has been demonstrated to be an early event and a potential therapeutic target in liver cancer development [21]. As reported, both the liver-specific knockout of Mst1/2 or Sav1 and the transgenic overexpression of YAP in mice expanded liver size and ultimately induced HCC, revealing a significant role of the Hippo-YAP signaling pathway in hepatocarcinogenesis [22, 23]. Previous studies have indicated an extensive nuclear localization of YAP in majority of CCA tissues [24, 25]. However, until now, the exact role of YAP in the CCA progression and the mechanisms still remain unknown.

In the present study we demonstrated that YAP is upregulated in CCA and its overexpression increases the oncogenic potential of CCA. Our study provided mechanistic evidence that YAP exhibits its oncogenic activity by increasing gankyrin expression via miR-29c and IGF1-induced AKT activation.

## RESULTS

### Up-regulation of YAP is associated with CCA poor prognosis

**Figure 1:**
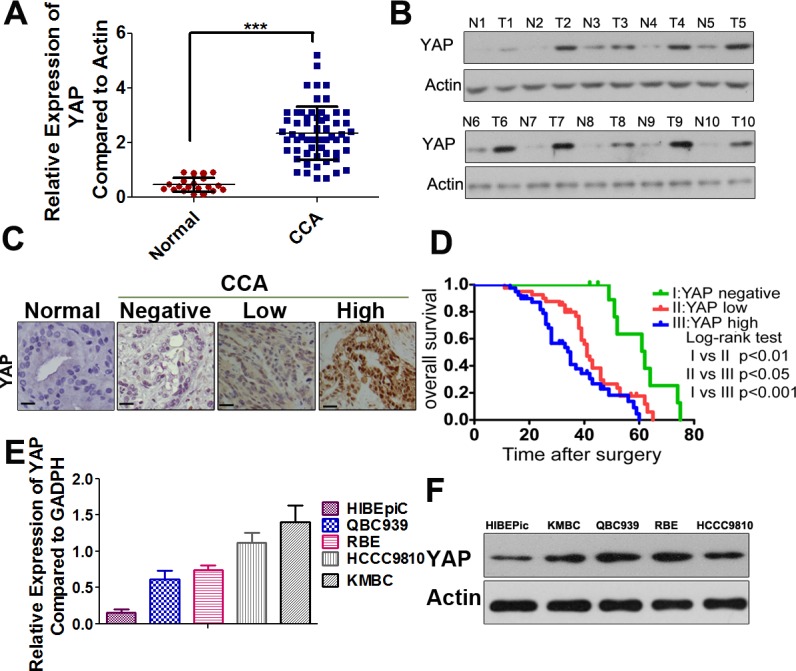
YAP is highly expressed in CCAs and predicts a poor prognosis **A.** YAP mRNA levels were significantly increased in a large percentage of human CCA tissues compared with normal bile duct tissues determined by qRT-PCR. ***, *P* < 0.001. **B.** Representative images of Western-blot assays in a subset of fresh frozen tissues confirmed the overexpression of YAP in human CCAs compared with normal tissues. T: tumors; N: normal tissues. **C.** Representative IHC staining of YAP in normal bile duct sample, YAP-negative (N: no positive YAP staining), YAP-low (L: below the median value of the integrated optical density) and YAP-high (H: above the median value of the integrated optical density) CCA samples were shown, black scale bar stands for 25 μm. **D.** The Kaplan-Meier method was used to determine the survival of 90 patients with CCA and log-rank test to compare survival among the YAP-negative, YAP-low and YAP-high groups. **E.** Relative YAP mRNA expression levels in HIBEpiC and CCA cell lines by qRT-PCR. **F**. Western blotting analysis of YAP in HIBEpiC and four CCA cell lines were performed. Actin was used as internal control.

Our qRT-PCR results demonstrated that YAP mRNA expression was increased in CCA samples compared to the paired non-malignant samples (Figure 1A). The qRT-PCR data were further confirmed by Western blot analysis (Figure 1B). Next, we detected the expression and subcellular localization of YAP protein by IHC in 90 cases of CCA and 25 specimens of nonneoplastic tissues. The results showed that YAP expression was detected in 85 (94%) CCA specimens, whereas only 4 (16%) of the non-malignant samples yielded positive YAP expression. YAP mainly showed positive expression in the nuclei of tumor cells, and was present to a lesser extent in the cytoplasm (Figure 1C). As accumulation of nYAP proteins is indicative of activation of YAP, the CCA patients were divided into three groups according to different status of nYAP expression (the cutoff for the definition of subgroups was the median value): the nYAP-negative group (*n* = 11), nYAP-low group (*n* = 40), and nYAP-high group (*n* = 39). Clinical association analysis by the chi-square test showed that nYAP expression in CCA was significantly associated with histological differentiation, TNM stage, lymph node metastasis and distant metastasis (Supplementary Table S1). According to the Kaplan-Meier method, we also found that the overall survival (OS) time in the patients with high-nYAP expression was significantly shorter than those with negative and low-nYAP expression (Figure 1D). To confirm the independent prognostic significance of nYAP, the expression of nYAP and those relative clinicopathological characteristics were further investigated in multivariate analysis. The data demonstrated that the expression of nYAP was an independent prognostic factor. With regard to other clinicopathological characteristics, only TNM stage showed significant prognostic influence for overall survival (Supplementary Table S2). Furthermore, the higher expression level of YAP protein and mRNA were found in CCA cell lines compared with that in HIBEpiC cells (Figure 1E and 1F).

### Silencing YAP inhibits CCA cell proliferation, cell cycle progression and tumorigenicity

**Figure 2:**
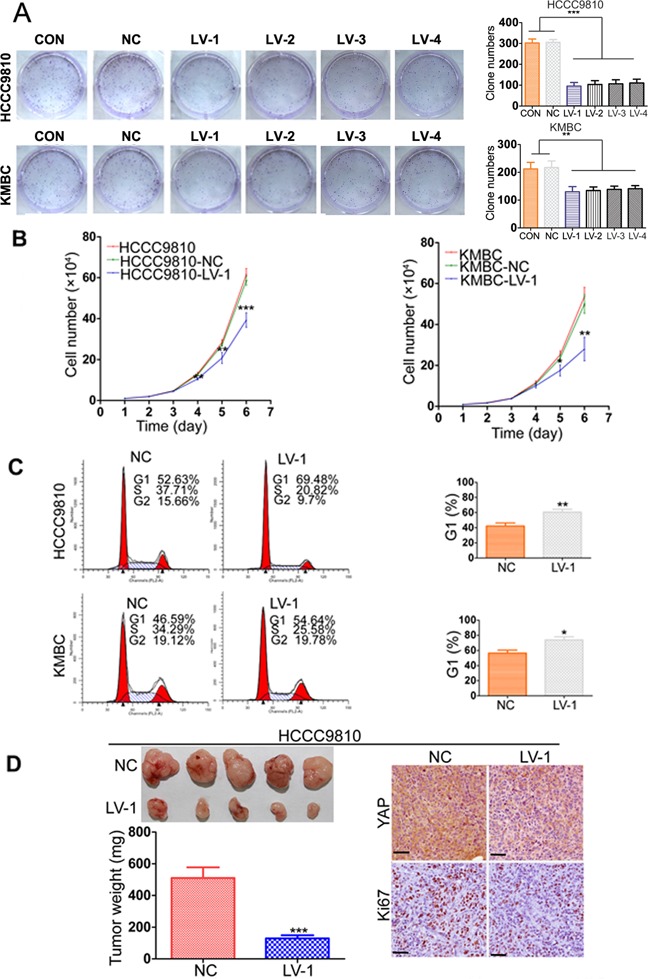
YAP knockdown inhibits CCA tumor growth both *in vitro* and *in vivo* **A.** Representative images of foci formation assays were shown in the left panels; the number of foci was counted as shown in the right panels. **B.** Growth curves for the indicated CCA cells (CON, NC, LV-1) were measured by direct cell counting. **C.** The cell cycle distribution of HCCC9810 and KMBC (NC and LV-1) cells were analyzed (left panel). Silencing YAP induced G1 cell cycle arrest (right panel). **D.** YAP knockdown reduced HCCC9810 cell xenograft tumor growth in nude mice. YAP and Ki-67 expression were examined by IHC staining. Scale bar stands for 25μm. CON, control group without any infection; NC, infected with negative lentivirus; LV, infected with Lenti-shRNA YAP. Experiments were done three times and data are presented as mean±SD. *P < 0.05; **P < 0.01; ***P < 0.001.

To investigate the role of YAP in CCA progression, we introduced Lenti-shRNA targeting YAP into CCA cells. YAP expression was remarkably decreased by Lenti-shRNA1 (LV-1) and moderately reduced by other three shRNAs (LV-2, 3, and 4), compared to the control shRNA (Supplementary Figure 1A). The downregulation of YAP protein expression was confirmed by Western blotting (Supplementary Figure 1B). The colony formation assays suggested that the capacities of CCA-LV cells to form foci were notably impaired compared with the controls (Figure 2A). In the growth curve assays, silencing YAP expression significantly suppressed the cell growth in the HCCC9810 and KMBC cell lines and the difference of cell number showed statistical significance from the fourth day (Figure 2B). We then performed cell cycle analysis and demonstrated that YAP knockdown arrested the cells at G1 phase (Figure 2C). Apoptosis assay was also carried out, but no significant apoptosis was detected after YAP knockdown in CCA cells (Supplementary Figure 2C). We further evaluated the effects of YAP knockdown on the growth of CCA xenograft tumors in nude mice, which were established by subcutaneously injecting HCCC9810-LV-1 cells and HCCC9810-NC cells into the flank, respectively. The time of tumor appearance was delayed in the HCCC9810-LV-1 group (14.50 ± 2.07 days) compared to the HCCC9810-NC group (8.37 ± 1.59 days). Compared with the control group, YAP knockdown led to smaller tumor size, lighter tumor weight, and decreased the expression of Ki-67 in the IHC analysis (Figure 2D).

### YAP overexpression promotes CCA cell proliferation and tumorigenicity

Next, we stably transfected two CCA cell lines, RBE and QBC939 with the YAP plasmid, and ectopic expression of the YAP in the cells was confirmed by western blotting and qRT-PCR (Supplementary Figure 2A and 2B). The growth curve assays showed that CCA-YAP cells grew much faster than the control cells, and statistical difference could be found from the fourth day (Supplementary Figure 2C). Colony formation assays yielded a higher number and larger colonies in the CCA-YAP cells compared to the control cells (Supplementary Figure 2D). Furthermore, we established a xenograft tumor mouse model by subcutaneously injecting QBC939-YAP cells and vector-transfected cells into the flank, respectively. We observed an earlier tumor formation in the overexpression group (5.12 ± 0.99 days) than that in the vector group (9.12 ± 1.80 days). The tumor size and weight were increased in the YAP overexpressed group compared to the vector group (Supplementary Figure 2E). The IHC results showed an increased expression of Ki-67 in QBC939-YAP group (Supplementary Figure 2E).

### YAP promotes CCA cell migration and invasion *in vitro* and metastasis *in vivo*

**Figure 3:**
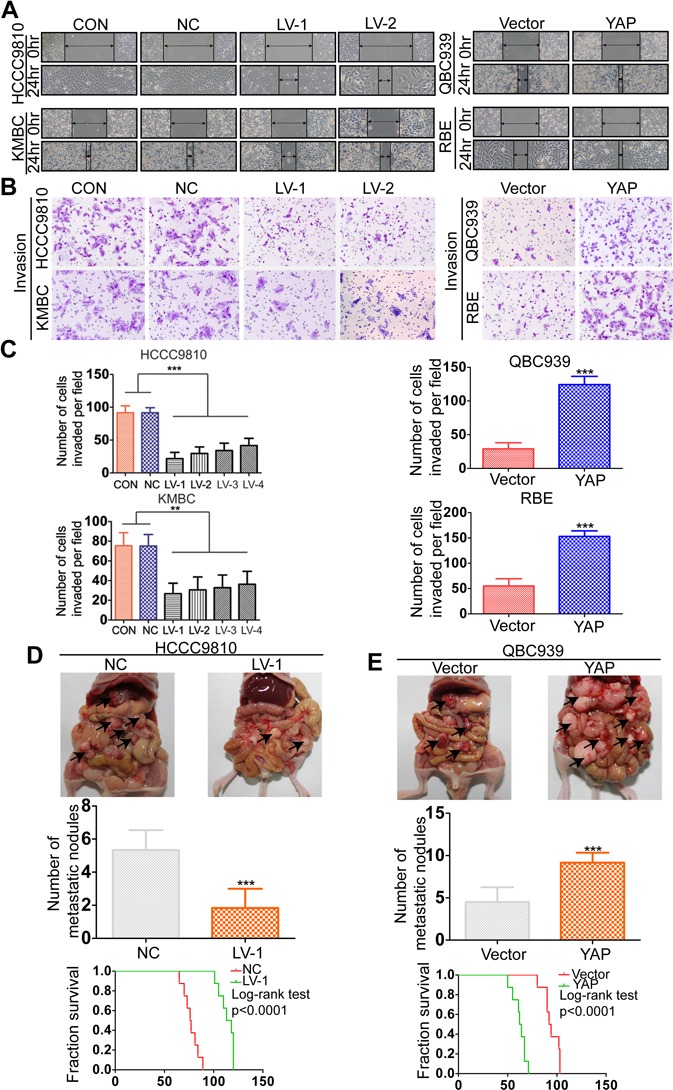
YAP promotes CCA metastasis both *in vitro* and *in vivo* **A.** Wound-healing assay showed that overexpressing YAP promoted CCA cells migration, whereas silencing YAP inhibited migration. Representative images were taken at 0 and 48 hours after scratching. **B.** Representative images of invasion assays for the CCA cell lines. **C.** The number of invaded cells was counted in different cell lines. **D.** The multiple tumor masses (black arrows) formed by the HCCC9810-NC cells were much more than that by HCCC9810-LV-1 cells (top and middle panel). The median survival time of the nude mice zenografted with HCCC9810-NC or HCCC9810-LV-1 cells were 77 and 113 days, respectively (bottom panel). **E.** The multiple tumor masses (black arrows) formed by the QBC939-vector cells were much less than that by QBC939-YAP cells (top and middle panel). The median survival time of the nude mice zenografted with QBC939-vector cells or QBC939-YAP cells were 94 and 62 days, respectively (bottom panel). Experiments were done three times and data are presented as mean±SD. **P < 0.01; ***P < 0.001.

YAP has been demonstrated to be associated with metastasis in a variety of malignant tumors [26–29]. Therefore, we then investigated whether YAP could increase the cell motility of CCA. In the wound-healing assay, YAP knockdown cells acquired slower closure of the scratched “wound” compared to the control cells. Conversely, YAP overexpression enhanced the abilities of CCA cells to cover the scratched “wound” (Figure 3A and Supplementary Figure 3A). The transwell migration and invasion assays showed that overexpresion of YAP significantly increased the migration and invasion capacities of RBE and QBC939 cells. In contrast, silencing YAP expression markedly decreased cell migration and invasion abilities of HCCC9810 and KMBC cells (Figure 3B–3C and Supplementary Figure 3B). Furthermore, we evaluated the role of YAP in tumor metastasis *in vivo* by injecting CCA cells into the peritoneal cavity of nude mice and monitoring the lethality over a 120-day period. Results of necropsy revealed that the number of metastatic nodules in the QBC939-YAP group was increased, compared to that in the vector group; however, the number of metastatic nodules in the HCCC9810-LV-1 group was significantly decreased, compared to that in the HCCC9810-NC group (Figure 3D and Supplementary Figure 3D). The QBC939-YAP group had a shorter OS time than the vector group, whereas the HCCC9810-LV-1 group had a longer OS time than the HCCC9810-NC group. We also found that tumors extensively colonized the visceral organs in the QBC939-YAP group (Figure 3E and Supplementary Figure 3D).

### YAP could induce epithelial-mesenchymal transition in CCA

**Figure 4:**
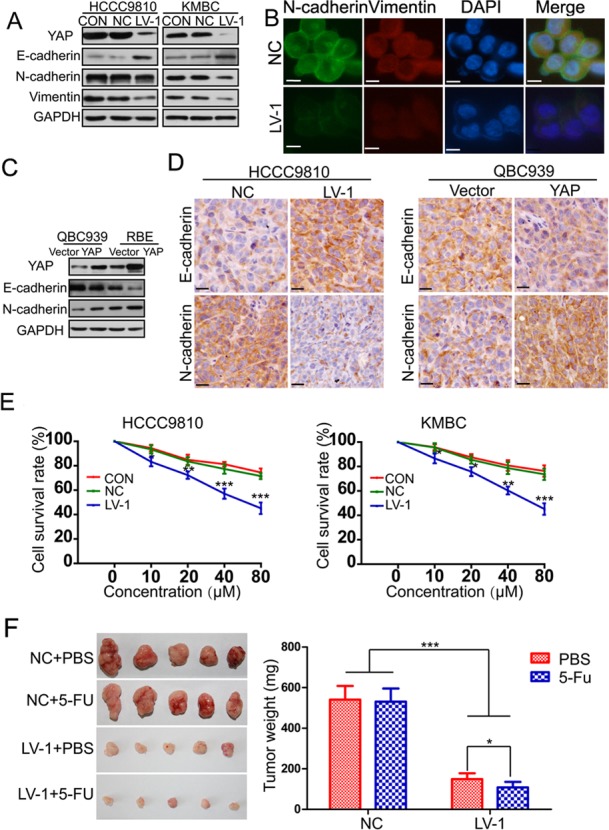
YAP induces epithelial-mesenchymal transition **A.** The expression of YAP, E-cadherin, N-cadherin and Vimentin was evaluated by Western blotting in HCCC9810 and KMBC cells (CON, NC, LV-1). **B.** E-cadherin and N-cadherin protein expression and subcellular localization were determined by immunofluorescence in QBC939 cells. Scale bar stands for 10μm. **C.** The expression of YAP, E-cadherin, N-cadherin and Vimentin was also detected by Western blotting in QBC939 and RBE cells stably transfected with YAP or control vector. **D.** The expression of E-cadherin and N-cadherin was detected by immunohistochemistry in xenograft tumor tissues from HCCC9810-NC, HCCC9810-LV-1, QBC939-vector and QBC939-YAP cells. Scale bar stands for 25μm. **E.** The sensitivity of HCCC9810 and KMBC cells to 5-FU was significantly enhanced after YAP knockdown. **F.** Effects of YAP knockdown on drug sensitivity of HCCC9810 cell xenograft tumors in nude mice. All data are the mean±SD of three separate experiments. *P < 0.05; **P < 0.01; ***P < 0.001.

It has been reported that EMT exists in a variety of malignant tumors of epithelial origin and is closely associated with invasion and metastasis [30]. Therefore, we investigated the effect of YAP on EMT by examining the expression patterns of epithelial and mesenchymal markers. Results from western blot showed that the epithelial marker E-cadherin was increased, whereas the mesenchymal markers N-cadherin and vimentin were decreased in HCCC9810-LV-1 and KMBC-LV-1 cells compared to the controls (Figure 4A). Immunofluorescent staining further confirmed the above-mentioned results in QBC939 cells (Figure 4B). An opposite expression patterns of E-cadherin and N-cadherin were detected in RBE-YAP and QBC939-YAP cells compared with the vector controls (Figure 4C). Moreover, we observed that YAP knockdown tumors exhibited elevated expression of E-cadherin and decreased expression of N-cadherin, whereas decreased expression of E-cadherin and increased expression of N-cadherin were found in YAP overexpressed tumors (Figure 4D). EMT has been reported to not only regulate cellular motility, but also lead to cell death resistance [30]. We next investigated whether YAP influenced the sensitivity of CCA to chemotherapeutic drugs. The data indicated that YAP knockdown increased sensitivity of CCA to 5-FU both *in vitro* and *in vivo* (Figure 4E and 4F).

### YAP increases the expression of gankyrin through microRNA-29c (miR-29c) and IGF1-induced AKT activation

**Figure 5:**
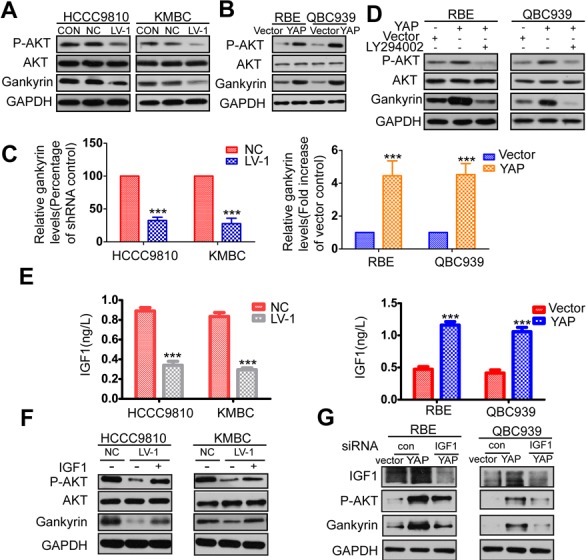
YAP increases gankyrin expression through AKT activation **A.** Relative expressions of AKT, p-AKT and gankyrin were evaluated by western blot in YAP konckdown and control CCA cells. **B.** Relative expression of AKT, p-AKT and gankyrin were evaluated by western blot analysis in YAP overexpressed and control cells. **C.** The mRNA level of gankyrin in YAP knockdown or overexpression cells compared to the control cells. **D.** Western blot analysis demonstrated that the AKT inhibitor LY294002 could effectively decreased expressions of p-AKT and gankyrin induced by YAP. **E.** ELISA analysis of IGF1 production in YAP knockdown or overexpression cells compared with the control cells. **F.** HCCC9810-LV-1 and KMBC-LV-1 cells were serum-starved, stimulated with IGF1, and immunoblotted for AKT, p-AKT and gankyrin. **G.** RBE-YAP and QBC939-YAP cells were transfected with IGF1 siRNA, relative expressions of IGF1, p-AKT and gankyrin were detected by Western blotting. All data are the means±SD of three separate experiments. ***P < 0.001.

We next wished to gain insight as to the mechanism by which YAP promoted CCA growth and metastasis. Signaling pathways involved in tumorigenesis and metastasis that might be activated by YAP were analyzed by examining the expression of phosphorylated forms of protein kinase B (AKT), extracellular signal-regulated kinase (ERK), and c-Jun N-terminal kinases (JNK) using Western blot assay (Figure 5A–5B and Supplementary Figure 4A). As we have previously reported, Gankyrin is crucial for CCA carcinogenesis and metastasis by activating IL-6/STAT3 signaling pathway through down-regulating Rb [31], we also detected the expression of gankyrin (Figure 5A–5B). Results indicated that silencing YAP reduced the expression of p-AKT and gankyrin, whereas YAP overexpression increased them (Figure 5A–5B). Moreover, the protein expression level of gankyrin was further confirmed in the xenografts by IHC analysis (Supplementary Figure 4B). We next investigated the effects of YAP on gankyrin transcription by qRT-PCR. The data demonstrated that YAP overexpression increased the mRNA level of gankyrin, whereas YAP knockdown decreased it (Figure 5C). As a transcription co-activator, YAP generally interacts with transcription factors to regulate the expression of target genes by binding to their promoters [6–10]. To explore the transcription factors responsible for YAP-induced gankyrin expression, mRNA levels of gankyrin were examined after siRNA-mediated inhibition of the known YAP binding partners in CCA cells. Other than siRNAs targeting YAP, only the TEAD4-specific siRNA led to a comparable reduction in gankyrin transcript and protein levels (Supplementary Figure 5A and 5B). Otherwise, silencing TEAD4 abolished YAP-induced transcript and protein expression of gankyrin in QBC939 cells (Supplementary Figure 5C and 5D). Then CHIP was performed to detect whether YAP directly increased gankyrin expression by binding to its promoter in HCCC9810 cells, but no positive result were gained (Supplementary Figure 5E). Importantly, co-IP assays showed that there was no direct protein interaction between YAP and Gankyrin (Supplementary Figure 5F). These results suggested that other factors might be involved in YAP-induced transcriptional activation of gankyrin. Li et al. demonstrated that the activation of PI3K-AKT signaling pathway by growth factor stimulation and Ras activation increased the expression of gankyrin [32]. Based on the above-mentioned fact, we hypothesized that YAP might increase the expression of gankyrin by activating PI3K-AKT signaling pathway. As expected, the result showed that the PI3K inhibitor LY294002 could effectively reduce expression levels of p-AKT and gankyrin protein in CCA-YAP cells (Figure 5D). YAP has been reported to directly increase the transcription of AXL to activate AKT [33]. Therefore, we next test whether AXL was required for the effects of YAP on gankyrin. We silenced AXL in YAP-overexpressing cells, but no apparent change of gankyrin expression was detected (Supplementary Figure 5G). Previous study reported that YAP could activate the kinases AKT by suppressing PTEN via miR-29c [34]. Therefore, We inhibited miR-29c using microRNA inhibitor in YAP-overexpressing cells to detect if miR-29c was required for the effects of YAP on gankyrin. The results showed that inhibiting miR-29c increased the expression of PTEN and reduced the expression of gankyrin and p-AKT (Supplementary Figure 5H). Moreover, CHIP assay further demonstrated that miR-29c was also a direct target of YAP in CCA cells (Supplementary Figure 5I). However, the expression of p-AKT and gankyrin are likely regulated by other factors as well because they were reduced but not completely abrogated by inhibiting miR-29c. YAP has also been reported to participate in the regulation of IGF1, which can activate AKT [35, 36]. We next explore the role of IGF1 on YAP-induced upregulation of gankyrin through AKT activation by loss-and gain-of-function. Data showed that YAP overexpression could increase the mRNA and protein levels of IGF1, whereas YAP knockdown could reduce them (Figure 5E and Supplementary Figure 6). Furthermore, IGF1 treatment rescued levels of p-AKT and gankyrin, which were decreased by YAP knockdown (Figure 5F). On the contrary, silencing IGF1 reduced the expression of p-AKT and gankyrin, which were increased by YAP overexpression (Figure 5G).

### Gankyin upregulates YAP at transcriptional level and is responsible for YAP-induced oncogenic activity

**Figure 6:**
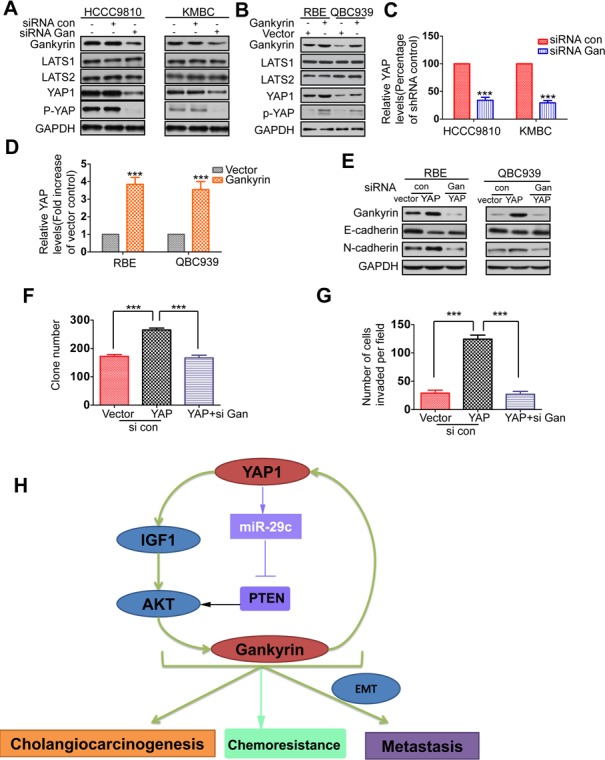
Gankyin upregulates YAP at transcriptional level and is responsible for YAP-induced oncogenic activity **A.** Relative expressions of gankyrin, LATS1, LATS2, YAP and p-YAP were detected by western blot in gankyrin knockdown and control cells. **B.** Relative expressions of gankyrin, LATS1, LATS2, YAP and p-YAP were detected by western blot analysis in Gankyrin overexpressed and control cells. **C.**-**D.** The mRNA level of YAP in gankyrin knockdown or overexpression cells compared to the control cells. **E.** RBE-YAP and QBC939-YAP cells were transfected with gankyrin siRNA, relative expressions of gankyrin, E-cadherin, N-cadherin were detected by Western blotting. **F.**-**G.** Colony formation and invasion assays were done for the QBC939-YAP cells following transfection with gankyrin siRNA. **H.** Schematic presentation of the mechanism underlying YAP-facilitated cholangiocarcinogenesis and metastasis. The results are presented as mean±SD from three independent experiments. ***P < 0.001.

As Gankyrin/IL-6 signaling shares a longstanding association with CCA carcinogenesis [28], we investigated whether gankyrin could conversely activate YAP signaling to form a feedback loop. Interestingly, the data showed that gankyrin knockdown did markedly reduce YAP protein level in CCA cells, whereas gankyrin overexpression increased it. We next investigated whether gankyrin increases YAP phosphorylation and cellular localization. We found a lower level of YAP phosphorylation (p-YAP) in gankyrin knockdown cells and a higher level of p-YAP in gankyrin overexpression cells compared to the controls (Figure 6A–6B). However, the protein level of LATS1, LATS2, and the ratio of p-YAP to total YAP were similar in gankyrin knockdown or overexpression cells compared with the controls. Furthermore, YAP nuclear localization was not affected by overexpressing gankyrin in QBC939 cells (Supplementary Figure 7A). Recent studies showed that YAP protein could be regulated by proteasomal degradation [37, 38]. Therefore, we tested whether gankyrin increases YAP expression by increasing protein stabilization. When RBE cells were treated with the protein synthesis inhibitor cycloheximide (CHX), YAP protein was unstable, with a half-life of approximately 1h. However, gankyrin overexpression did not stabilize YAP protein (Supplementary Figure 7B and 7C). We then carried out qRT-PCR to detect whether gankyrin increases YAP at transcriptional level. The data showed that gankyrin overexpression increased the mRNA level of YAP, whereas gankyrin knockdown decreased it (Figure 6C–6D). Aforementioned results aroused our interest to determine whether gankyrin played a role in mediating the oncogenic activity of YAP. We introduced siRNA and plasmid to knockdown or overexpress gankyrin expression in CCA cells. The results revealed that silencing gankyrin supressed YAP overexpression-enhanced proliferation and invasion in QBC939 cells, and the concomitant up-regulation of E-cadherin and down-regulation of N-cadherin expression in QBC939 and RBE cells (Figure 6E, 6F and 6G). Conversely, gankyrin overexpression restored YAP knockdown-induced inhibition of cell proliferation and invasion in HCCC9810 cells (Supplementary Figure 7D and 7E). Meanwhile, it reversed the expression of E-cadherin and N-cadherin in HCCC9810 and KMBC cells (Supplementary Figure 7F). To determine whether these results were reproducible in tumorigenesis or metastasis *in vivo*, we then silencing gankyrin in mice, which were subcutaneously or intraperitoneally injected with QBC939-YAP cells. Data showed that tumor growth and metastasis were significantly decreased by gankyrin knockdown (Supplementary Figure 7G).

### Combination of YAP and gankyrin or p-AKT exhibits improved prognostic accuracy for patients

**Figure 7:**
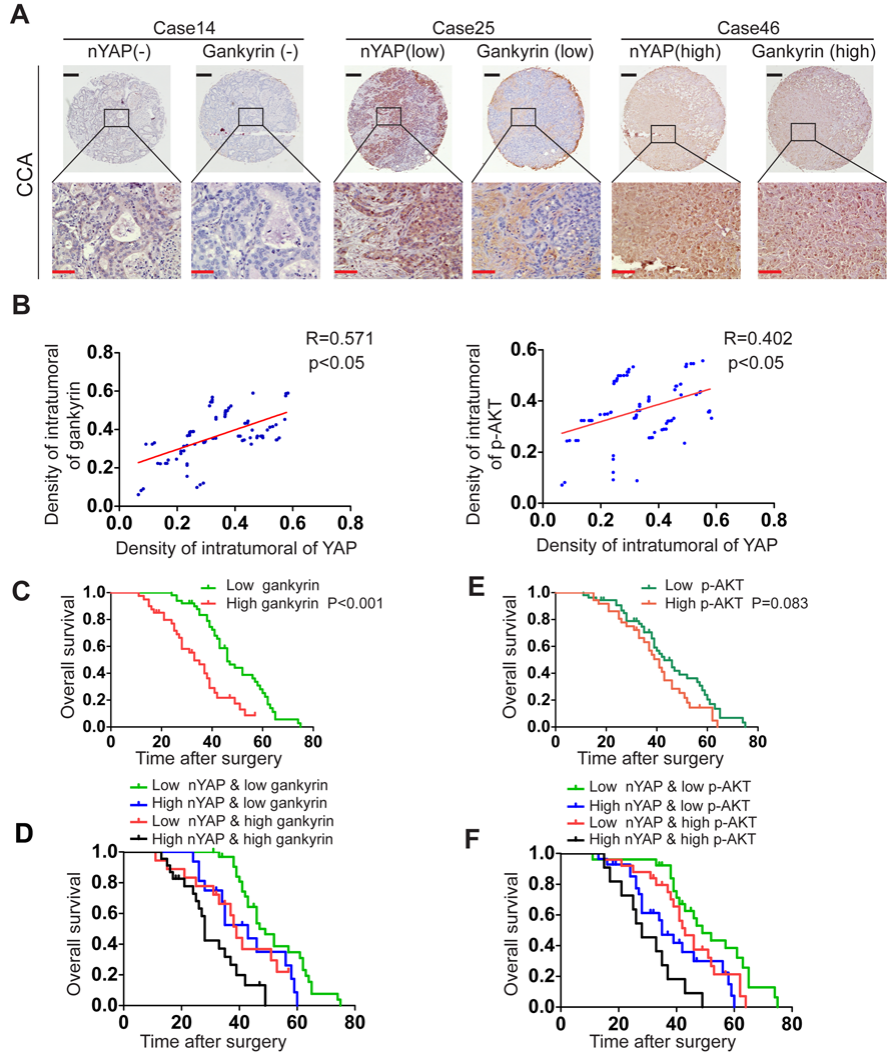
Combination of nYAP and gankyrin or p-AKT improves prognostic accuracy for CCA patients **A.** Representative view of IHC analysis of YAP and gankyrin expression in 90 CCA tissues. Black and red scale bar stands for 100μm and 25μm respectively. **B.** Correlation between YAP expression and gankyrin or p-AKT level was analyzed by Pearson correlation analysis. **C.** Kaplan-Meier's analysis of gankyrin expression in CCA patients after curative resection. **D.** The Kaplan-Meier analysis of concurrent nYAP and gankyrin expression with overall survival. E, Kaplan-Meier's analysis of p-AKT expression in CCA patients after curative resection. F, The Kaplan-Meier analysis of concurrent nYAP and p-AKT expression with overall survival.

Given that the reciprocal relationship between YAP and gankyrin described as above, we further analyzed the expression levels of nYAP and gankyrin in tissue microarray including 90 CCA specimens. Results showed a positive correlation between nYAP and gankyrin in CCA patients (Figure 7A and 7B). Otherwise, nYAP expression was also positively correlated with the levels of p-AKT (Figure 7B). Accumulating evidence demonstrates that, with proper combination, multiple markers might be more accurate than any one alone for predicting the prognosis of patients. In this study, we found that gankyrin overexpression was associated with poor prognosis, and CCA patients with reduced nYAP and gankyrin expression levels exhibited longer OS than those patients with elevated nYAP expression or increased expression of gankyrin (Figure 7C–7D). Although the elevated expression of p-AKT alone was not a predictor factor (*p* = 0.083) of CCA, the combination of nYAP and p-AKT increased the prognostic value, as compared to nYAP or p-AKT alone (Figure 7E–7F).

## DISCUSSION

Up to now, there is evidence connecting YAP with the tumorigenicity of a wide spectrum of human tumors [11–20]. Previous studies have revealed that YAP was mainly expressed in the cell nuclei in CCA patient specimens [24, 25]. In the current study, we found that YAP protein was detected in 85 (94%) CCA specimens and mainly showed positive expression in the nuclei of tumor cells, whereas Tao et al. has found that YAP protein was almost ubiquitously detected in the nucleus (98.4%) [24]. Although the explanation for this discrepancy is unclear, we thought there were mainly two reasons. First, specimens used for IHC analysis in our study were both from extrahepatic and intrahepatic CCA samples, whereas the specimens used in Tao's study were totally from intrahepatic CCA. Interestingly, we found that the CCA tissues with YAP protein ubiquitously detected in the cytoplasm were almost all form extrahepatic CCA specimens, which might be due to the clinical pathology difference between extrahepatic and intrahepatic CCA patients. Second, the different background of the CCA patients, including the human species (our study was investigating the Chinese patients) and etiology of CCA may be the other reason. Furthermore, We found that nYAP expression was significantly associated with CCA histological differentiation, TNM stage, and metastasis. The positive expression of nYAP was an independent predictor of short overall survival of CCA patients, as confirmed by the Kaplan-Meier curves and multivariate Cox proportional hazards regression analysis. Taken together, the results suggested that YAP might be involved in CCA pathogenesis.

YAP has been reported to induce EMT, boost proliferation, and promote tumorigenicity and metastasis in a context-dependent manner [26–29, 39–42]. In our study, we demonstrated that YAP overexpression significantly promoted cell growth and tumor formation in nude mice, while YAP knockdown efficaciously suppressed the cell growth, tumorigenicity, and induced G1 cell cycle arrest. In addition, the results derived from *in vitro* cell migration, invasion assay, and *in vivo* metastasis assay confirmed that YAP increase the ability of CCA invasion and metastasis. As EMT has been reported to be associated with tumor invasion and metastasis, we determined whether YAP enhanced the invasion and metastasis of CCA by inducing EMT [30]. As expected, YAP overexpression resulted in the decrease expression of epithelial markers and increase of mesenchymal markers, whereas silencing YAP reversed them. In view of the important role that EMT played in cell death resistance, we examined whether YAP increased the sensitivity of CCA to chemotherapeutic drugs [30]. The data showed that down-regulation of YAP enhanced the sensitivity of CCA to 5-FU both *in vitro* and *in vivo*. These findings were consistent with the results in a recent study, which reported that inhibition of YAP expression sensitized HCC cells to doxorubicin [43]. Taken together, YAP could potently facilitate tumorigenesis and metastasis in many respects throughout the progression of CCA.

Gankyrin, the p28 component of the 26S proteasome, has been identified as an oncoprotein in a variety of malignant tumors [44–48]. We recently reported that gankyrin could promote CCA tumor growth and metastasis through activation of IL-6/STAT3 signaling [31]. In the present study, we found that YAP could increase gankyrin expression at the level of protein and mRNA. As a transcriptional co-activator, YAP generally interacts with transcription factors to regulate the expression of target genes [6–10]. To our disappointment, we found no DNA-binding sites on the promoter sequence of gankyrin. However, results showed that YAP could increase gankyrin expression through PI3K-AKT signaling, which were consistent with a recent study indicating that the activation of PI3K-AKT signaling increased gankyrin expression [32]. Some of the targets controlled by YAP have been reported to be involved in the activation of PI3K-AKT signaling, such as AXL, CCN2, miR-29c, and IGF-1 [33–35, 49]. We further found that YAP increased gankyrin expression through miR-29c- and IGF1-mediated AKT activation. Otherwise, our data indicated that YAP exhibited the oncogenic activity and induced EMT though increasing gankyrin expression in CCA. More interestingly, we found that gankyrin could in turn increase YAP expression at transcriptional level in CCA. Taken together, these results indicated that a positive feedback loop, consisting of YAP, miR-29c, IGF1, AKT, and gankyrin, was involved in the progression of CCA (Figure 6H). Furthermore, the results from the IHC analysis in CCA specimens further confirmed the close connection between nYAP and gankyrin or p-AKT. Moreover the predictive range of nYAP expression levels combined with gankyrin or p-AKT was more sensitive than that of nYAP alone for OS, strongly suggesting that the abovementioned regulatory circuit were recapitulated in clinical patients with CCA.

In summary, the present paper indicated that YAP was overexpressed in human CCA cell lines and patient specimens, and nYAP was an independent prognostic marker for overall survival of CCA. Overexpression of YAP significantly promoted CCA tumor growth and metastasis, whereas silencing YAP reduced CCA tumorigenesis and metastasis both *in vitro* and *in vivo*. Our study demonstrated a previously unrecognized pathway in YAP-induced cholangiocarcinogenesis and metastasis, which suggested therapeutic targets, including YAP, IGF1, AKT, and gankyrin, in CCA prevention and treatment. Further characterization of YAP may result in the discovery of therapeutic targets for better clinical management of CCA.

## MATERIALS AND METHODS

### Cell lines and regents

The human CCA cell line KMBC, HCCC9810 and RBE were obtained from Shanghai Bioleaf Biotech Co., Ltd. (Shanghai, China). QBC939 cell line was given as a present by Professor SG Wang, from the Third Military Medical University of China. The normal human intrahepatic biliary cell line (HIBEpiC) was obtained from ScienCell Research Laboratories (Carlsbad, CA). CCA cell lines were cultured in RPMI1640 or DMEM supplemented with 10% fetal bovine serum (FBS). LY294002, CHX and IGF1 was purchased from Sigma-Aldrich. IGF1 ELISA Kit was obtained from Abcam.

### ELISA

Supernatants from CCA cells were collected and IGF1 ELISA was subsequently performed using IGF1 ELISA Kit and following the manufacturer's instructions.

### Immunofluorescence (IF)

CCA cells were seeded onto glass slides, and fixed in 4% paraformaldehyde after complete adherence. The slides were then permeabilized with 0.5% Triton X-100 for 15 minutes and blocked using normal goat serum for 30 minutes. The primary antibodies were added and incubated at 4°C overnight. The slides were washed with PBS for three times and incubated with Alexa Fluor 488-conjugated secondary antibodies at room temperature for an hour. After that, 4′6-diamino-2-phenylindole (DAPI) was added to stain the cell nuclei and images were captured.

### *In vivo* spontaneous metastasis assay

All mice were obtained from the laboratory animal center of the Chinese academy of sciences, Shanghai. The experimental protocol was reviewed and approved by the Committee on the Use of Live Animals in Teaching and Research of the Harbin Medical University, Harbin, China. To evaluate the peritoneal metastasis, BALB/c nude mice, 6-8 weeks of age, were used in the experiments (*n* = 8/group). 5×10^6^ CCA cells were inoculated into the intraperitoneal cavity. Mice were killed at 4 months after injection. Solid tumors and organs were removed and examined.

### Statistical methods

Statistical analysis was performed using the GraphPad Prism software package (v. 4.02; GraphPad Prism Software Inc, San Diego, CA) or SPSS 16.0 software (SPSS, Chicago, IL, USA). All values were expressed as mean ± SD. Analysis of variance (ANOVA) and a Student's t-test were used to evaluate statistical significance. The association of nuclear YAP (nYAP) expression with CCA patient's clinicopathological characteristics and gankyrin or p-AKT level were evaluated using the χ^2^-test. The Kaplan-Meier method was used to evaluate the probability of patient survival, and the significant difference was determined using the log-rank test. Univariate and multivariate survival analysis was performed to evaluate the independent factors using the Cox proportional hazards regression model. A value of less than 0.05 (*P* < 0.05) was used for statistical significance.

Detailed description of Patients and Methods can be found in the online Supporting Information.

## SUPPLEMENTAL MATERIAL TABLES AND FIGURES



## Abbreviations

CCA: cholangiocarcinoma
YAP: Yes-associated protein
nYAP: nuclear YAP
TEAD: TEA domain family member
EMT: epithelial-to-mesenchymal transition
OS: overall survival
IHC: Immunohistochemical
AKT: protein kinase B
ERK: extracellular signal-regulated kinase
JNK: c-Jun N-terminal kinases
IF: Immunofluorescence
qRT-PCR: quantitative reverse transcription polymerase chain reaction
siRNA: small interfering RNA
5-fluorouracil: 5-FU
