# Supplementary material for: YAP is a critical oncogene in human cholangiocarcinoma
Source: Oncotarget. 2015 May 8;6(19):17206–20. doi: 10.18632/oncotarget.4043 (PMC4627302; doi:10.18632/oncotarget.4043)
Supplement: Supplementary file 1 [file oncotarget-06-17206-s001.pdf]

YAP is a critical oncogene in human cholangiocarcinoma

Supplemental Material

Patients and Tissue samples

A retrospective cohort of 90 CCA patients, who underwent routine surgical procedures at the First Affiliated Hospital of Harbin Medical University between 2005 and 2012, were included in this study. The pathologic diagnosis of CCA was performed according to World Health Organization criteria. The grade of differentiation was evaluated according to the classification proposed by Edmondson and Steiner. The disease stage of each patient was classified according to the sixth edition of the TNM classification system published by the International Union Against Cancer. Clinicopathological information about the patients was obtained from patient records, and was listed in Table SI. All samples used in this paper were approved by the Committees for Ethical Review of Research at the First Affiliated Hospital of Harbin Medical University.

Establishment of Knockdown Cells and Construct design of lentiviral-mediated small hairpin RNA (Lenti-shRNA) targeting YAP and Gankyrin

Lenti-viral containing short hairpin RNAs (shRNA) against YAP and gankyrin were manipulated according to the manufacturer's protocol as described previously [1]. We used the lentiviral vector pGCSIL-GFP-Negative (GeneChem) as a negative control to evaluate

nonspecific responses caused by heterologous shRNA. Stable transfectant clones were determined by quantitative real-time PCR, and western blot was used to detect their expression levels of YAP and Gankyrin. The sequences for shRNAs targeting the YAP or Gankyrin gene are as follows:

YAP-LV-1: 5'-GACTCAGGATGGAGAAATTTA-3';

YAP-LV-2: 5'-GCCACCAAGCTAGATAAAGAA-3';

YAP-LV-3: 5'-CGACCAATAGCTCAGATCCTT-3';

YAP-LV-4: 5'-CAGGTGATACTATCAACCAAA-3';

Gankyrin-KD-1: 5'-GGTTGGTCTCCTCTTCATA-3'

Gankyrin-KD-2: 5'-CAGCTTGGATTTATTCTTA-3'

Gankyrin-KD-3: 5'-GTTACTTGTTCGAAGCTTA-3'

Gankyrin-KD-4: 5'-GTTGGATGGTGTGCTCTAA-3'

Overexpression of YAP and Gankyrin in CCA Cells

YAP plasmid and empty vector were constructed and purchasded from GeneChem (Shanghai, China). The Gankyrin plasmid and empty vector were provided as gifts from Prof. Fujita Jun (Department of Clinical Molecular Biology, Kyoto University, Japan). Plasmids were transfected into CCA cells by using Lipofectamine 2000 (Invitrogen) according to the manufacturer’s instruction. CCA cells transfected with empty vector were used as controls. Stable expressing clones were selected and the expression level was evaluated.

RNA-interference

The CCA cells were transfected with siRNAs by using Lipofectamine 2000 (Invitrogen, Carlsbad, CA, USA). Control siRNA (sc-37007) and siRNA targeting gankyrin (sc-72186) , IGF1 (SC-37193) were purchased from Santa Cruz Biotechnology. TEAD4 (107036) and MicroRNA 29c inhibitors (000587) were purchased from Ambion. SiRNA flexiplate was purchased to dectect the transcription factors responsible for YAP-induced gankyrin expression. The siRNA sequences were as follows:

SMAD1 TTAGCTCAGTTCCGTAACTTA;

SMAD3 AAGAGATTCGAATGACGGTAA;

SMAD4 CCCTGTTAAACAGTAGTTGTA;

RUNX2 CAGAAGCTTGATGACTCTAAA;

TEAD1 CGCCGCTTCATTGCACATTCA;

TEAD2 CTCCAGGTGGTGACAAACAGA;

TEAD3 AAGGTCCTCACTGTTTGCATA;

Western blot

Procedure for western blot was performed routinely, as reported previousl [2].The following antibodies against YAP, β-catenin, E-cadherin, AKT, p-AKT (Ser473), ERK, p-ERK (Thr202/Thr204), JNK and p-JNK (Thr183/Thr185) were obtained from Cell Signaling Technology. Primary antibodies against IGF1, N-cadherin and vimentin were purchased from

Abcam. Antibody against gankyrin and GAPDH were obtained from Santa Cruz Biotechnology.

Coimmunoprecipitation (co-IP) assay

The cells were lysed in 400 μl lysis buffer (10 mM Tris-HCl, pH 8.0, 100 mM NaC1, 10 mM EDTA and 0.5% NP-40) containing protease inhibitors. The lysates were incubated with 15μl of anti-Gankyrin monoclonal antibody at 4°C for 2 h. The complex were then precipitated with 15μl protein A-Sepharose and incubated for 4h at 4°C with gentle rotation. After washing with lysis buffer, the beads were boiled with loading buffer and analyzed by Western blotting. Normal mouse IgG was used as a negative control.

Taqman Real-Time PCR Analysis

TaqMan real-time PCR was performed as described using commercially available primers designed against human YAP, Gankyrin and GAPDH [1]. Total RNA was extracted from cells and tissues using Trizol (Invitrogen, CA, USA). Real-time PCR was performed in triplicate with Taqman PCR Mix (Applied Biosystems) in the ABI Step-One system. The primers used in real-time PCR were as follows:

Forward primers:

Gankyrin: 5'-CAATCAAAATGGCTGTACTCCCTTA-3';

YAP: 5'-CCTCGTTTTGCCATGAACCAG-3';

IGF1: 5'-GCAGCCTTCCAACTCAATTATTTAA-3'

miR-29c promoter: 5' -TTCTGTTGACTC CTAGCA GCC-3'

CTGF promoter: 5'-CTTTGGAGAGTT TCAAGAGCC-3'

GAPDH: 5'-GAAGGTGAAGGTCGGAGTC-3';

Reverse primers:

Gankyrin: 5'-TGGTCCTTAGCATCTGGATTAGC-3';

YAP: 5'-GTTCTTGCTGTTTCAGCCGCAG-3';

IGF1:5'-GTAGAAGAGGTGTGAAGACGACATG-3'

miR-29c promoter：5'-GTGTAGCTGTAGTCAGA-3'

CTGF promoter：5'-CCTACATACAGTCACCTGTCT-3'

GAPDH: 5'-GAAGATGGTGATGGGATTTC-3';

Cell growth and Colony formation assay

For the cell growth assays, CCA cells (5-6x10^4 /well) were seeded in six-well plates. The number of viable cells was counted at different time points. For colony formation assays, five or six hundred cells of the HCCC9810, KMBC, QBC939, or RBE cell line were cultured in six-well plates for 14 days and then colonies were stained with 0.05% crystal violet for 30 min and counted.

Cell cycle analysis

Cells (4x10^5) were fixed in 70% ethanol for 1 h at 4˚C. Then the cells were washed twice with PBS and 10 mg/mL RNase A was added. After that propidium iodide was added to the tubes at a final concentration of 0.05 mg/ml, and the samples were incubated at 4˚C for 30 min in a dark

environment. The result was analyzed by flow cytometry (Beckman Coulter EPICSALTRA II).

Apoptosis assay

CCA cells (2 x10^5 /well) were cultured in six-well plates, and then were collected and washed twice with ice-cold PBS. Apoptosis was investigated by flow cytometry using Annexin V-PE Apoptosis Kit (Becton Dickinson, San Diego, CA) following the protocol.

Wound-Healing Assay

For the wound-healing assay, CCA cells were cultured in six-well plates until confluence and then scratched with a 10μL pipette tip. Then images were captured at 0 and 24h hours after scratching.

Migration and Invasion Assay

Cell motility and invasive capacites were investigated by way of transwell (BD Biosciences, San Jose, CA, USA) and Matrigel invasion (BD Biosciences), respectively. For transwell migration assay, 2-3x10^4 cells were seeded, whereas 3-4x10^4 cells were seeded for the invasion assay. The assay had been performed for 36 h at 37 °C, non-migrated or non-invaded cells were removed from the upper surface of the membrane. Cells migrated to the underside of the membrane were fixed and stained with 0.5% crystal violet, and then were counted under an optical microscope. Each experiment was repeated at least three times.

Chromatin immunoprecipitation assay

CCA cells were cross-linked by the addition of formaldehyde to a 1% final concentration, the chromatin was sonicated, and the immunoprecipitation was performed using 1 μg of YAP and IgG antibody. ChIP assays were performed using a commercially available ChIP assay kit (Simple ChIP Cell Signaling Technology) following the manufacturer's instructions. The sequence of primers for gankyrin used to amplify ChIP enriched DNA is available upon request. The mRNA level of gankyrin were evaluated by RT-PCR.

Construction of tissue microarrays and immunohistochemistry

CCA samples and specimens of nonneoplastic tissues were used to construct a tissue microarray (Shanghai Biochip Co., Ltd. Shanghai, China). Expression of YAP, gankyrin, p-AKT, Ki-67, E-cadherin, and N-cadherin in tumor tissues was detected by IHC analysis as described previously [1]. The density was evaluated by Image-Pro Plus v6.2 software (Media Cybernetics Inc, Bethesda, MD). For the reading of each antibody staining, a uniform setting for all the slides was applied. Integrated optical density of all the positive staining in each photograph was measured, and its ratio to total area of each photograph was calculated as density. For YAP density, the cutoff for the definition of subgroups was the median value. Samples were then segregated into two

groups for each analysis. The first group comprised samples in which YAP expression was above the median value (YAP-high group), and the second group comprised the rest of the samples (YAP-low group). Each data set was analyzed separately and consensus evaluation from at least two of the three investigators was considered acceptable.

Subcutaneous CCA Experiment

Male BALB/c (5-6 weeks old) mice (n=8/group) were injected subcutaneously in the flank with 2 $x10^6$ CCA cells suspended in phosphate buffered saline (PBS). The mice were observed over 4 weeks for tumor formation. The tumor apparence time, size and weight were evaluated. The experiments on drug sensitivity were separated into two groups (NC and LV-1). The dosage of 5-FU was 80mg/kg/day (using PBS for the control), administered on day 8 after injection. Mice were sacrificed at day 35 after posttransplantation. Tumor size and weight were evaluated. The experiments involving living mice was reviewed and approved by the Committee on the Use of Live Animals in Teaching and Research of the Harbin Medical University, Harbin, China.

Table S1 Correlation between YAP expression and clinicopathological characteristics of CCA patients

clinical characteristics	Total	nYAP expression		P-value
		Negative-Low	High	
All case	90	51	39	
Gender				
Male	49	28	21	0.921
Female	41	23	18	
Age				
≥60 years	44	27	17	0.379
<60 years	46	24	22	
Differentiation				
Well	36	25	11	
Moderate	25	16	9	0.013
Poor	29	10	19	
TNM stage				
I	32	23	9	
II	22	14	8	0.011
III	19	10	9	
IV	17	4	13	
Nodal metastasis				
pN0	43	29	14	0.048
pN1	47	22	25	
Distant metastasis				
M0	67	44	23	0.003
M1	23	7	16	

Table.S2 Univariate and multivariate analysis of factors associated with survival in CCA patients

Factors	Univariate P	Multivariate Analysis		
		HR	95%CI	P
Gender (Male vs Female)	0.421	—	—	—
Age (< 60 y vs ≥ 60 y)	0.362	—	—	—
Differentiation (P vs M/W)	0.292	—	—	—
Nodal metastasis (pN0 vs pN1)	0.986	—	—	—
Distant metastasis (M0 vs M1)	0.216	—	—	—
nYAP (Negative-Low vs High)	0.016	1.742	1.067 2.844	0.026
TNM stage (I / II vs III / IV)	0.007	1.854	1.143 3.008	0.012

OS indicates overall survival; HR, hazard ratio; CI, confidence interval.

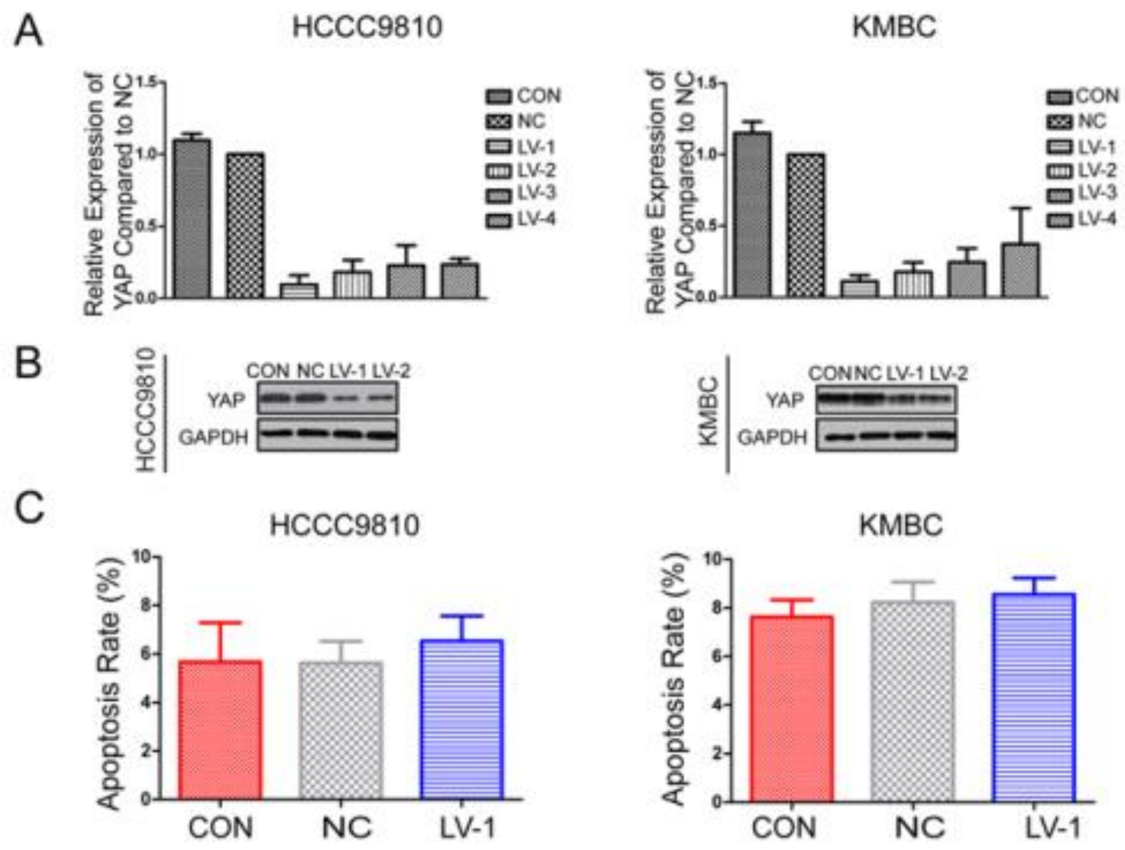

Supplementary Figure 1: A, The expression of YAP was lower after knockdown by LV-shRNA than the control sequences. **B,** Downregulation of YAP protein expression was confirmed by Western blotting. **C,** The apoptosis rate were quantified and presented as means±SD.

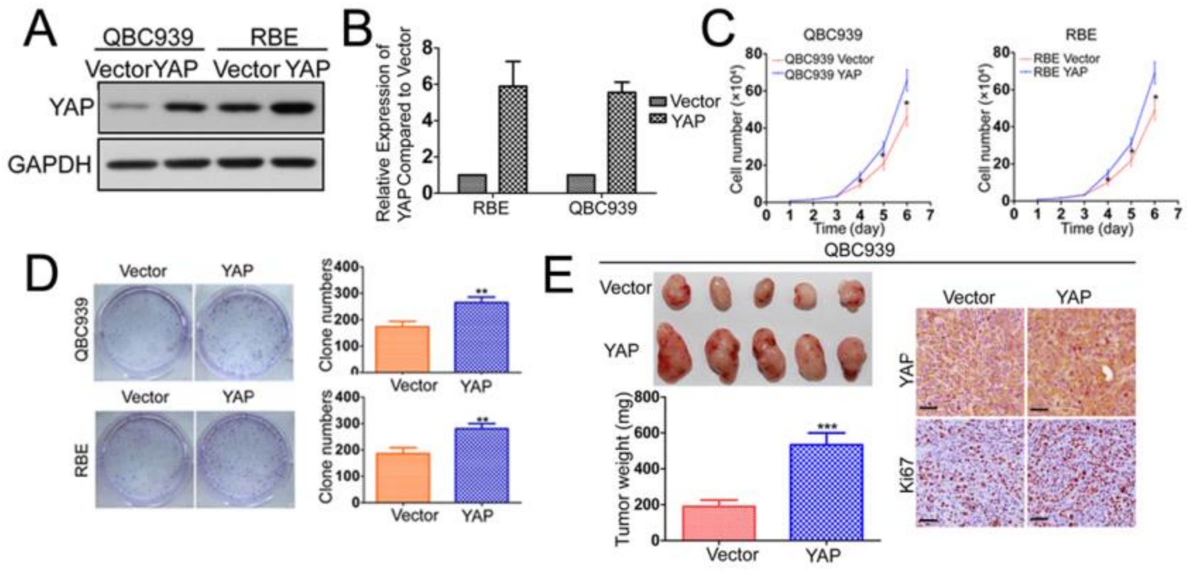

Supplementary Figure 2: YAP overexpression promotes CCA cell proliferation and tumorigenicity. A-B, Western blot and Real-time PCR analysis showing ectopic expression of YAP in YAP-transfected cells. CON, control group without any infection; NC, infected with negative lentivirus; LV-1, infected with Lenti-shRNA1 YAP. **C,** Growth curves of YAP overexpressed or vector cells were measured by direct cell counting. **D,** Representative images of foci formation assays were shown in the left panels. The number of foci was counted as shown in the right panels. **E,** YAP overexpression increased QBC939 cell xenograft tumor growth in nude mice. YAP and Ki-67 expression were examined by IHC staining. Scale bar stand for 100μm. All data are the mean±SD of three separate experiments. *P < 0.05; **P < 0.01; *** P < 0.001.

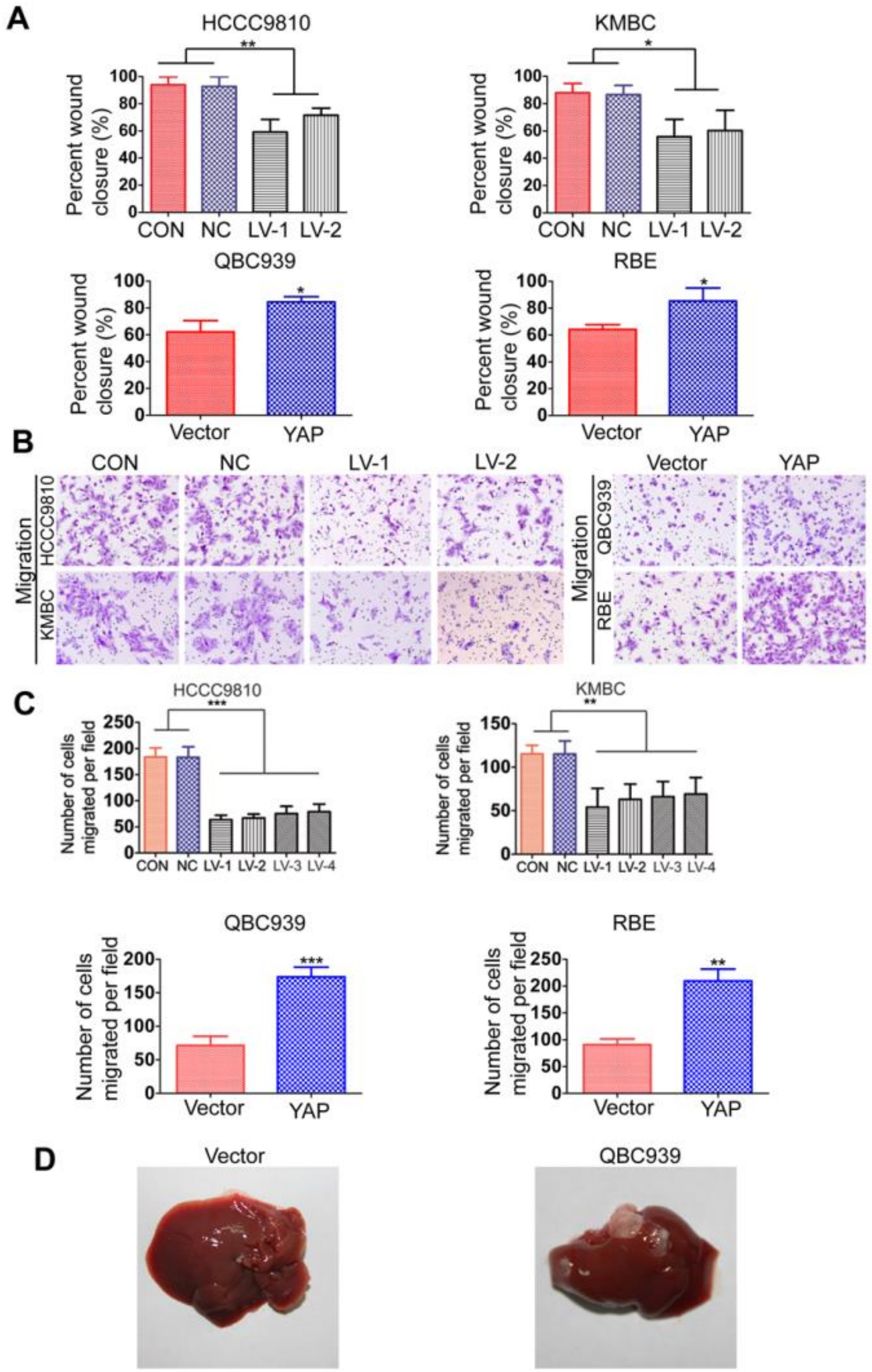

Supplementary Figure 3: A, The widths of the gaps in wound-healing assays were measured, and the results are shown as a bar graph. **B,**

Representative images of migration assays for the CCA cell lines. **C,** The number of migrated cells was counted. **D,** Representative images of tumor colonizing the visceral organs in the QBC939-YAP group. The results are presented as mean±SD from three independent experiments. * $P < 0.05$; ** $P < 0.01$; *** $P < 0.001$.

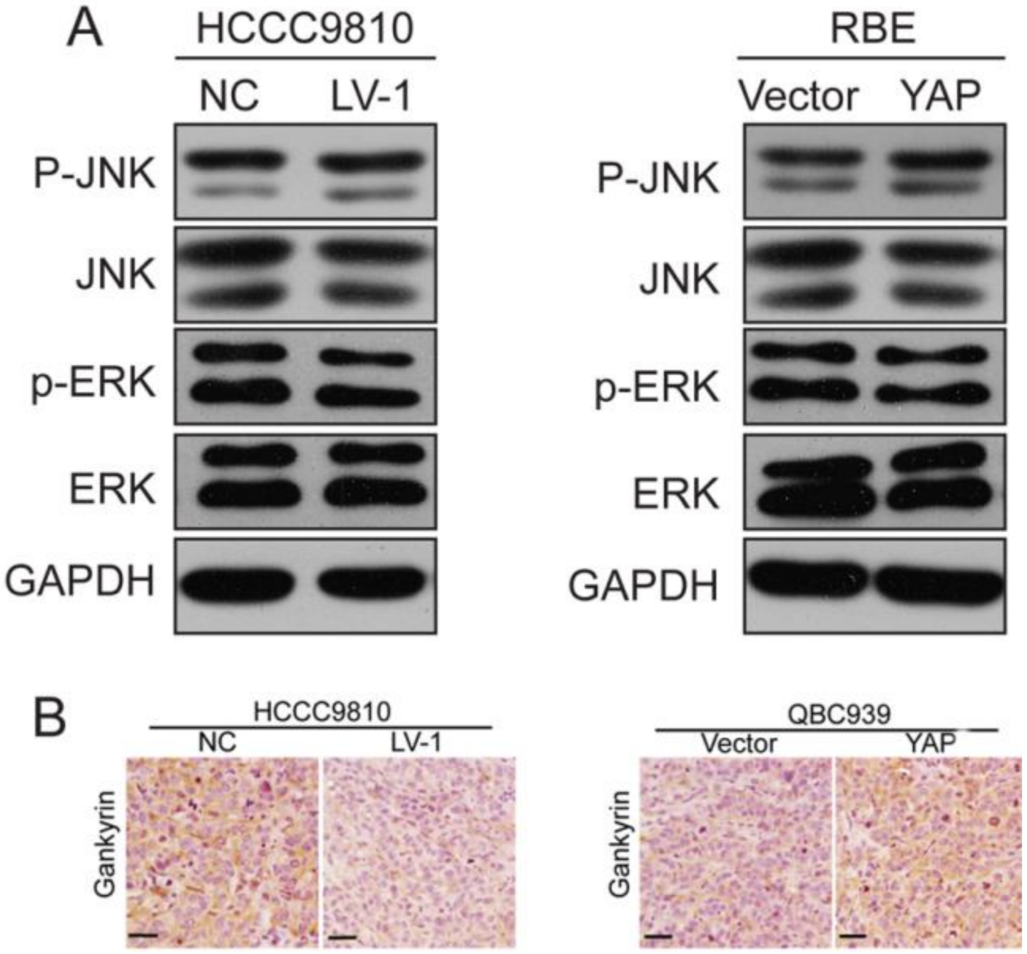

Supplementary Figure 4: A, Expressions of p-ERK, ERK, p-JNK, JNK and actin were determined by Western blot in HCCC9810 control and YAP knockdown cells (left panels); expressions of p-ERK, ERK, p-JNK, JNK and β-actin were determined by Western blot in RBE control and YAP overexpressed cells (right panels). **B,** The expression level of gankyrin was evaluated in the xenografts by IHC analysis. Scale bar stand for 100μm.

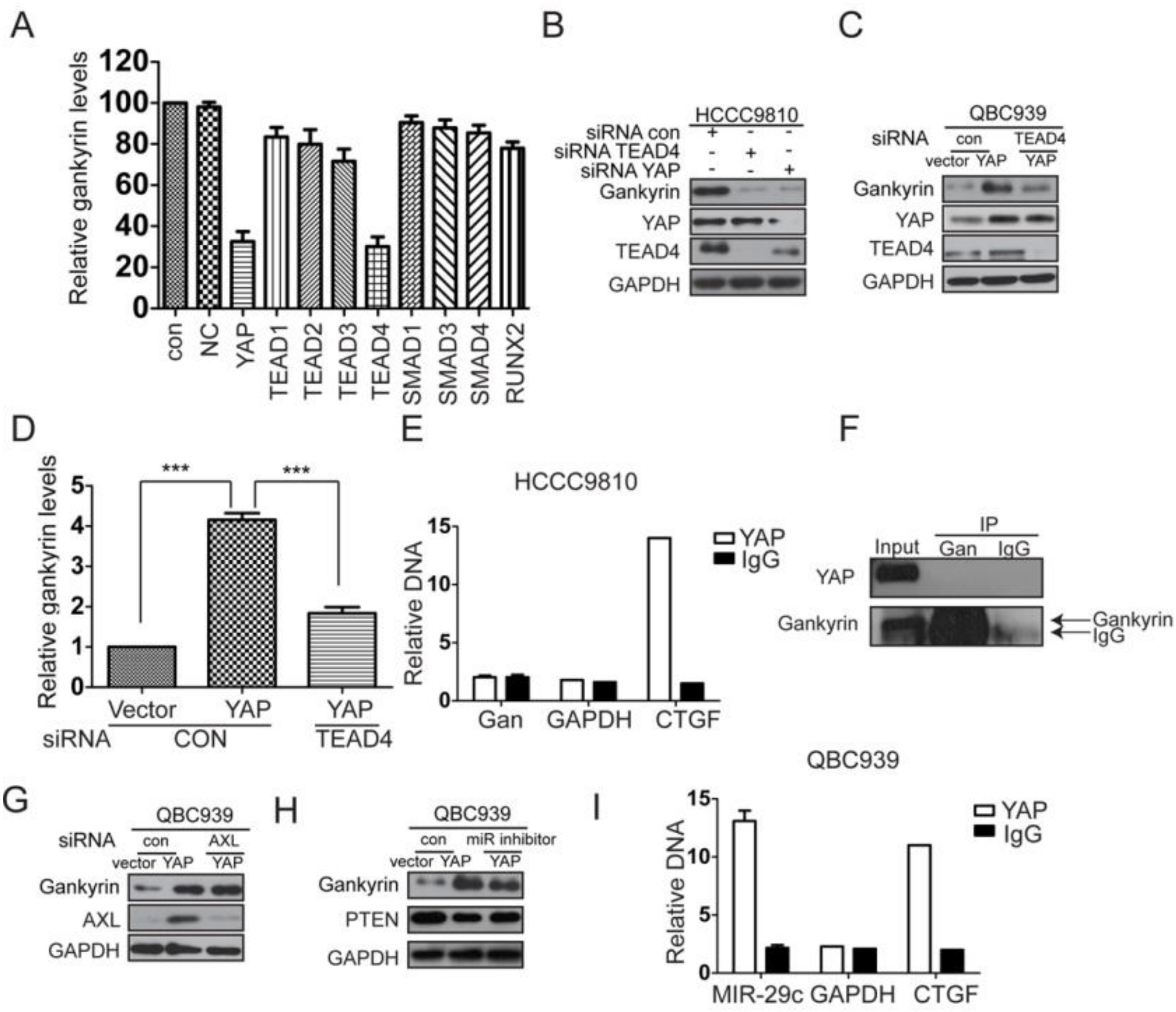

Supplementary Figure 5: A, RT-PCR of gankyrin mRNA after inhibition of YAP-interacting transcription factors. Untreated (CON) and scramble siRNA-transfected cells served as NC; YAP inhibition were used as positive controls. Untreated cell samples were used for calibration. **B,** Western blot analysis of YAP, gankyrin and TEAD4 in HCCC9810 cells after siRNA-mediated inhibition of YAP and TEAD4. **C,** QBC939-YAP cells were transfected with TEAD4 siRNA, relative expressions of gankyrin, YAP and TEAD4 were detected by Western

blotting. **D,** QBC939-YAP cells were transfected with TEAD4 siRNA, relative mRNA expressions of gankyrin were detected by real-time PCR. **E,** ChIP was performed to detect the interaction between YAP and the promoter of gankyrin using anti-YAP and anti-IgG antibodies. GAPDH and the known YAP target CTGF were shown as negative or positive control. **F,** Interaction of endogenous YAP and Gankyrin assessed by co-IP. **G,** QBC939-YAP cells were transfected with AXL siRNA, relative expression of gankyrin and AXL was detected by Western blotting. **H,** QBC939-YAP cells were transfected with miR inhibitor, relative expression of gankyrin, AKT, p-AKT and PTEN was detected by Western blotting. **I,** ChIP was performed to detect the interaction between YAP and the promoter of miR-29c using anti-YAP and anti-IgG antibodies. GAPDH and the known YAP target CTGF were shown as negative or positive control. *** $P<0.001$

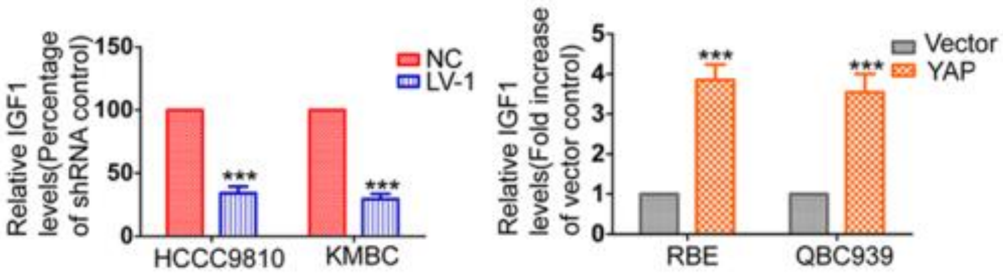

Supplementary Figure 6: A, The mRNA level of IGF1 in YAP knockdown or overexpression cells compared to the control cells. All data are the mean±SD of three separate experiments. *** P < 0.001.

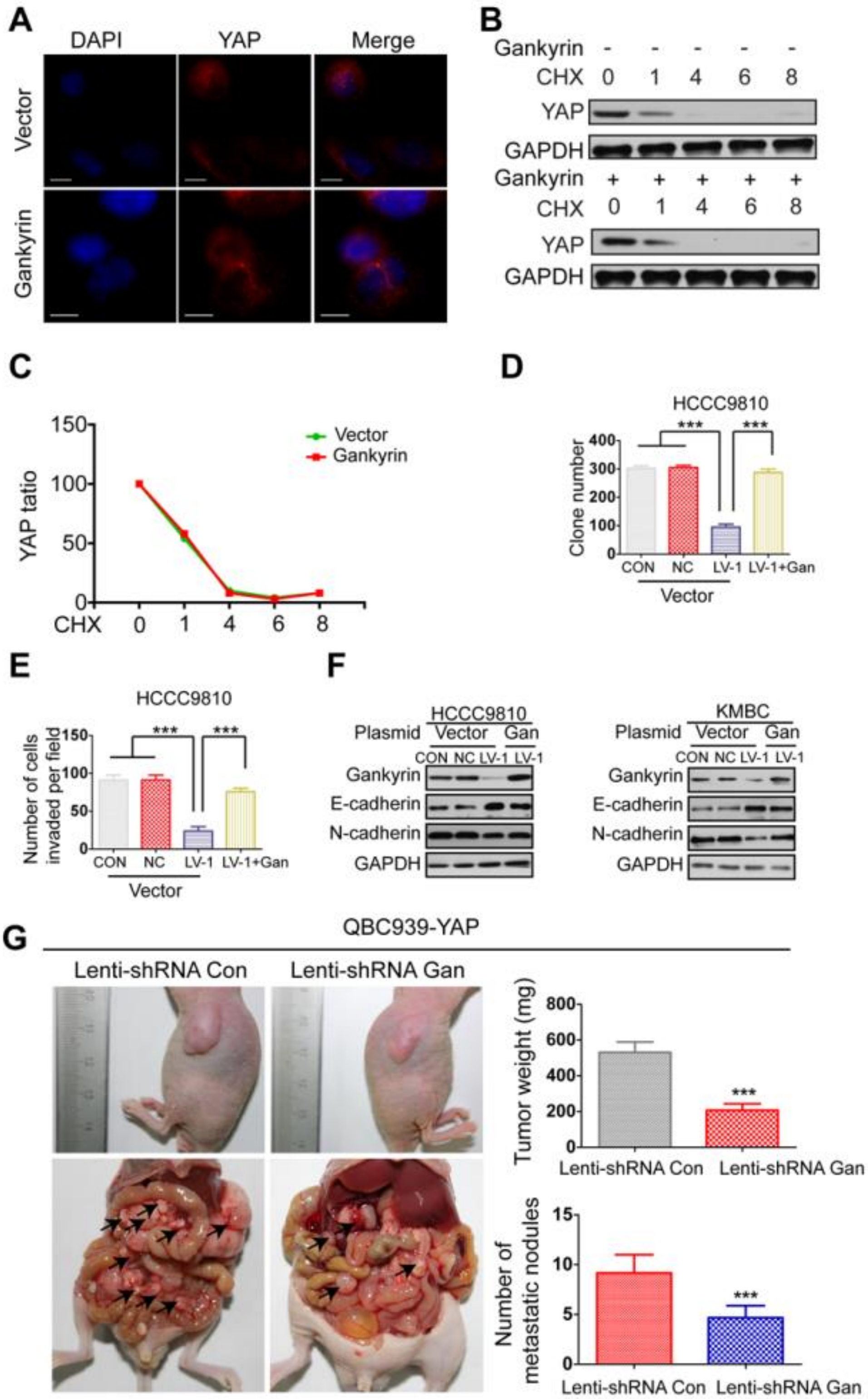

Supplementary Figure 7: Gankyrin is responsible for YAP-mediated oncogenic activity. A, YAP protein expression and

subcellular localization were determined by immunofluorescence in QBC939 cells stably transfected with gankyrin or control vector. Scale bar stands for 10 um. **B,** The level of YAP protein synthesis in RBE cells after the treatment of CHX for the indicated time. **C,** Relative YAP protein levels were quantified by YAP/GAPDH ratio. **D-E,** Colony formation and invasion assays were done for the HCCC9810 knockdown cells following with gankyrin overexpression. **F,** HCCC9810 knockdown and KMBC knockdown cells were transfected with gankyrin plasmid, the expression of gankyrin, E-cadherin, N-cadherin was detected by Western blotting. **G,** QBC939-YAP cells transfected with Lenti-mediated shRNA control or gankyrin were subcutaneously or intraperitoneally injected into nude mice. The tumor weight and number of metastasis nodules were evaluated. All data are the means±SD of three separate experiments. *** $P < 0.001$.

Supplementary references:

1. Zheng T, Hong X, Wang J, Pei T, Liang Y, Yin D, et al. Gankyrin promotes tumor growth and metastasis through activation of IL-6/STAT3 signaling in human cholangiocarcinoma. Hepatology. 2014;59:935-46.
